# Oculomotor Nerve Neuromyotonia: An Inflammatory Syndrome

**DOI:** 10.1212/WNL.0000000000209769

**Published:** 2024-08-12

**Authors:** Adam Handel, Sarosh R. Irani, Ashwini Oswal

**Affiliations:** From the Nuffield Department of Clinical Neurosciences (A.H., A.O.), University of Oxford, United Kingdom; and Department of Neurology (S.R.I.), Mayo Clinic, Jacksonville, FL.

A 68-year-old woman presented with a 1-year history of episodic, painless double vision. On examination, diplopia was triggered by sustained right lateral gaze. On subsequent return to neural gaze, the left eye remained ‘locked’ in adduction (Video 1). Impairments of abduction and vertical movements of the left eye were noted, consistent with a diagnosis of left oculomotor nerve neuromyotonia.^1^

10.1212/209769_Video_1 Video 1 : Left oculomotor neuromyotonia triggered by right lateral gaze. Episodes lasted up to 3 minutes and occurred at least 10 times daily. Tonic contraction of the left medial rectus leads to the left eye being fixed in an adducted position with little horizontal or vertical mobility.Download Supplementary Video 1 via http://dx.doi.org/10.1212/209769_Video_1

MRI revealed smooth enhancement of the left oculomotor nerve (Figure), which was unchanged on repeat scanning after 6 months but had resolved after 1 year. Investigations did not reveal a causative pathology, and the patient's symptoms resolved with carbamazepine. An inflammatory process was thought most likely based on the spontaneous resolution of the enhancement pattern.

**Figure:**
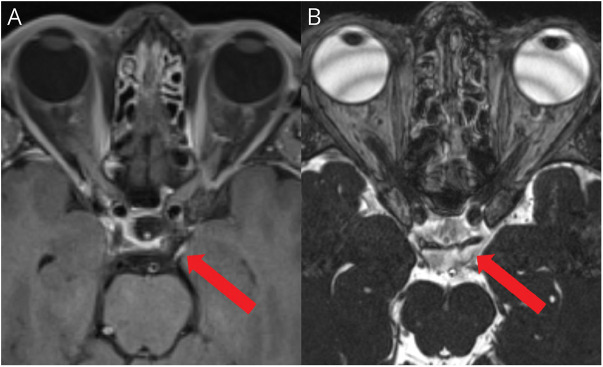
MRI Imaging Findings (A) Axial T1 postcontrast MRI demonstrating smooth enhancement of the cisternal portion of the left oculomotor nerve. (A) CSF filled dural cuff surrounds the nerve, which is susceptible to inflammatory, compressive, or neoplastic pathology.^2^ (B) The 3D constructive interference in steady state sequence is useful for visualizing abnormal signal within the nerve (red arrow).

Ocular neuromyotonia is a rare condition causing transient diplopia due to extraocular muscle spasm, often secondary to ephaptic transmission in a damaged ocular motor nerve. Common causes include cranial irradiation, but many cases are idiopathic.^1^

## Disclosure

A. Oswal has received research support from an MRC Clinician Scientist Fellowship (MR/W024810/1). A. Handel has received funding from the MRC (MR/X022013/1) and UCB Pharma. S.R. Irani has received honoraria/research support from UCB, BioHaven therapeutics, Immunovant, MedImmun, Roche, Janssen, Cerebral therapeutics, ADC therapeutics, Brain, CSL Behring, and ONO Pharma, receives licensed royalties on patent application WO/2010/046716 entitled “Neurological Autoimmune Disorders,” has filed 2 other patents entitled “Diagnostic method and therapy” (WO2019211633 and US app 17/051,930; PCT application WO202189788A1) and “Biomarkers” (WO202189788A1, US App 18/279,624; PCT/GB2022/050614), and has received research support from an MRC Senior Clinical Fellowship (MR/V007173/1), a Wellcome Trust Fellowship (104079/Z/14/Z), and the National Institute for Health Research (NIHR) Oxford Biomedical Research Centre (BRC).
